# Supplementary figures and images for: Temporal and spatial relationship between gluteal muscle Surface EMG activity and the vertical component of the ground reaction force during walking
Source: PLoS One. 2021 May 26;16(5):e0251758. doi: 10.1371/journal.pone.0251758 (PMC8153502; doi:10.1371/journal.pone.0251758)

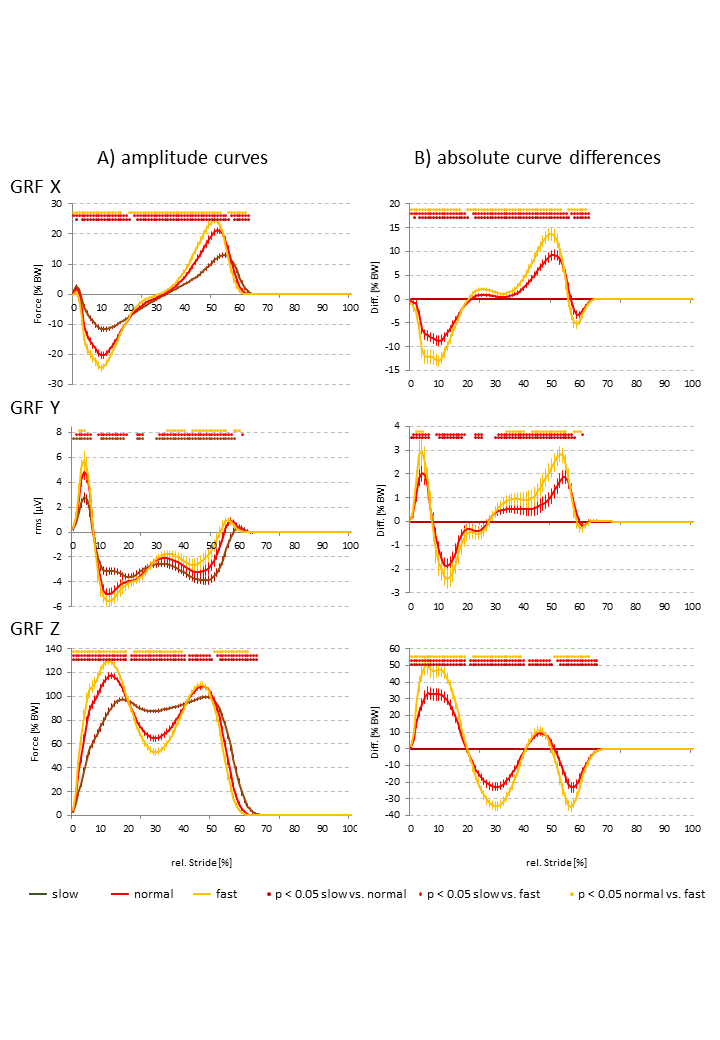

Supplement: S1 Fig — Grand averaged curves (A) and curve differences following normalization to the slow walking speed (B) for the GRF x, y, and z directions. Significant differences among the individual walking speeds are indicated by colored bars (p < 0.05; Bonferroni-Holm procedure). Data are given as mean values ± 95% confidence intervals. (TIF) [file pone.0251758.s001.tif]

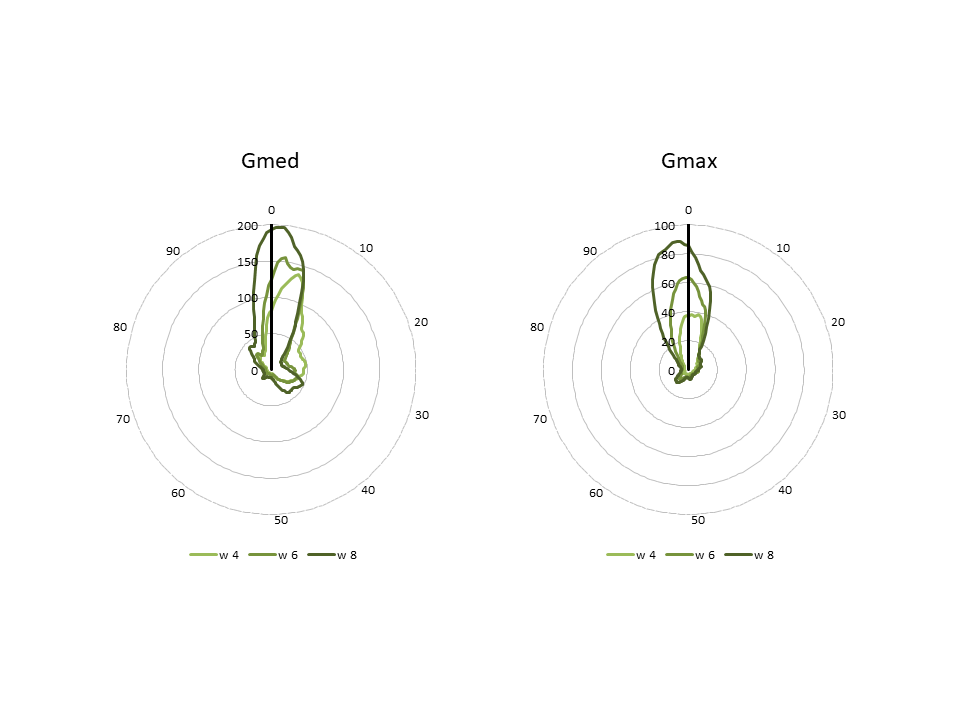

Supplement: S2 Fig — Electrodes for Gmed and Gmax were positioned at the recommended SENIAM positions (http://www.seniam.org). Data are given as mean values. (TIF) [file pone.0251758.s002.tif]

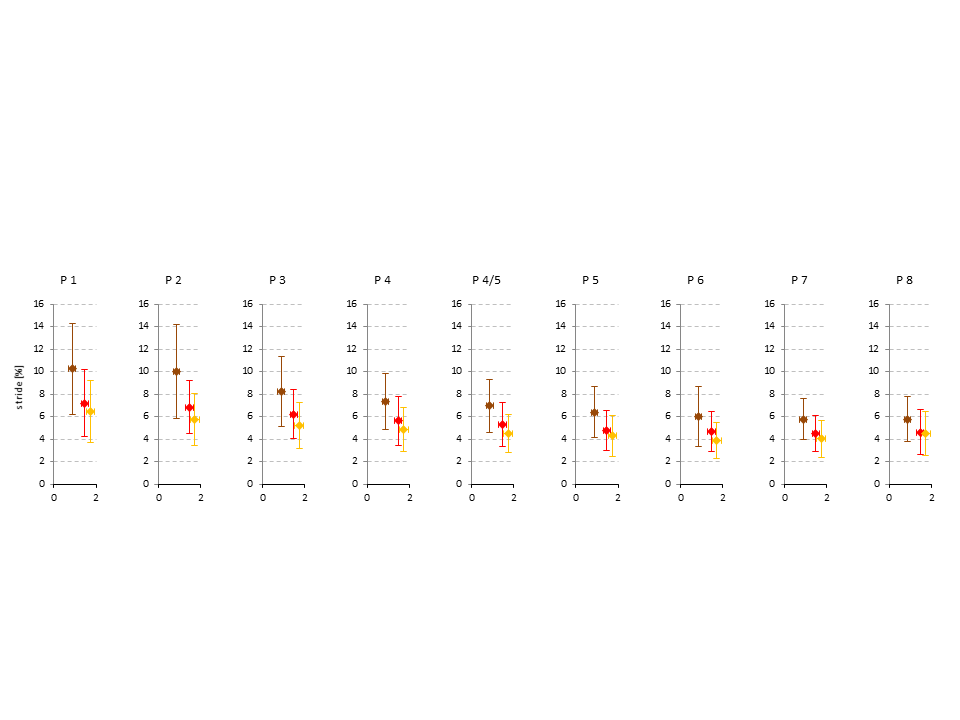

Supplement: S3 Fig — Data are calculated on a subject-individual basis and given as mean values ± SD. (TIF) [file pone.0251758.s003.tif]
